# Causes of admissions and mortality in patients of a wildlife rehabilitation centre in Bulgaria

**DOI:** 10.3897/BDJ.12.e123681

**Published:** 2024-07-01

**Authors:** Ivanka Asenova Lazarova, Gergana Nikolova Balieva, Ivailo Klisurov

**Affiliations:** 1 Trakia university, Stara Zagora, Bulgaria Trakia university Stara Zagora Bulgaria; 2 Green Balkans, Stara Zagora, Bulgaria Green Balkans Stara Zagora Bulgaria

**Keywords:** incidents, wild birds, wildlife rehabilitation, conservation

## Abstract

Wildlife rescue centres are specialised units with ecological, conservational and veterinary medical activities, which include treatment, rehabilitation, breeding and releasing rare and endangered wild animals into their natural habitat, as well as environmental education. These centres provide an opportunity to monitor ongoing ecological changes in wildlife, environmental pollution and emerging diseases. With the present study, we aimed to analyse the causes and conservation status of the largest wildlife rehabilitation centre in Bulgaria. A total of 18,720 patients, from 26 orders with various conservation statuses and different etiology, have been admitted to the rehabilitation centre for over 25 years. The summarised results showed that 40% of the patients were admitted with an unknown etiology and the proportion of anthropogenic causes was 18%. Natural factors related to incidents with wild animals were 32%, while a share of 10% of the wildlife which resided at the WRBC referred to a part of re-introduction programmes. This type of analysis of wildlife rehabilitation centres could provide useful information about the status of populations and ecosystems, as well as support conservation practices.

## Introduction

The International Union for Conservation of Nature (IUCN) created a Red List of Threatened Species. This list is a crucial measure of the condition of worldwide biodiversity. It contains data about the range, population size, habitat, ecology, exploitation and/or trade, threats,and actions required to safeguard plant and animal species. A Red List of threatened species was also prepared by region. The European list includes 15,000 species, with 1,677 facing extinction (IUCN 2022).

In response to threats to human and animal health, the term "conservation medicine" emerged (Kock 1996). It combined the implementation of several activities, including study of environmental health problems, emerging diseases, biological effects of various pollutants, consequences of environmental changes and others (Sleeman 2008).

According to Kock (1996), veterinary health care plays a crucial role in maintaining the overall health of wildlife. This involves activities such as diagnosing, preventing and treating animal diseases, as well as keeping a close watch on their health. Additionally, veterinary health care is responsible for ensuring the well-being of wild animals in captivity and preparing them for release into their natural habitat.

The rehabilitation and therapy of wild animals and birds have greatly developed in recent years. This has had a positive impact on their health, improved their welfare and helped to restore some endangered species (Negro et al. 2007). The wildlife rescue centres where wildlife in distress were admitted, have, as their primary objective, the goal to restore the health of wildlife specimens that have suffered accidents and prepare them for release back into the wild (Molina-Lopez et al. 2014). Rehabilitation centres play a crucial role in safeguarding biological diversity and ecosystems and, as a result, they are prevalent worldwide. The implementation of their activities varies depending on the availability of material and non-material resources (Klisurov 2012a). 

In France, there are over 45 licensed centres for rehabilitation of birds and other wild animals. These centres are located throughout the country and are mainly focused on protected species. The animals that are admitted to these centres have mostly suffered from accidents caused by environmental disasters, such as oil pollution or outbreaks of various diseases (Gourlay et al. 2014). In many regions of the US, wildlife rehabilitation is a widely accepted practice that greatly supports national policies in this area (Hanson et al. 2021). In Italy, there are 97 centres managed by public or private entities in close cooperation with local administrations (Dessalvi et al. 2021).

According to the European Parliament, the primary factors contributing to the loss of biodiversity are land-use changes, rising urbanisation, climate change, pollution and uncontrolled hunting. The conflict between humans and animals is causing the extinction and decrease in many species, numerous incidents that involve humans, some of which are fatal and significant economic losses (Nyhus 2016,Mekonen 2020, Nyhus 2016). As per a report published by the European Commission (Silva et al. 2018), instances of illegal activities against wildlife are increasing in the 21^st^ century. The nature of these activities varies depending on the motives, targets of the act and the methods employed.

Wildlife crime is considered one of the most severe and well-organised criminal activities, ranking alongside illegal acts such as counterfeiting, drug distribution and arms smuggling (Europol 2017). The consequences of this activity result in a decrease in biodiversity and the extinction of endangered animal species. With the present study, we aimed to analyse the causes for incidents amongst patients of the largest wildlife rehabilitation centre in Bulgaria. Our purpose was to determine the tendencies of their occurrence through the years.

## Material and methods

The present study analysed data on patients of the Wildlife Rehabilitation and Breeding Centre at Green Balkans - Stara Zagora (WRBC), which was established as a leading unit in wild animal rescue activities in Bulgaria.

The Centre's database for the period 1995-2019 was analysed through authorised personal access. Due to the specific activity of the WRBC, the patients were systematised, based on their conservation status and the protection of species, according to the current legal framework: Biological Diversity Act of Bulgaria, the International Union for the Protection of Nature, the Convention on the Protection of Wild European Flora and Fauna and Natural Habitats (Bern Convention), the Convention on the Conservation of Migratory Species of Wild Animals (Bonn Convention), Convention on International Trade in Endangered Species of Wild Fauna and Flora (CITES).

For the purpose of the study, we divided the patients according to the cause for their admission into two main groups: natural causes and anthropogenic causes leading to incidents with wild animals, which were as well divided into subcategories as per the established etiological factors.

In order to investigate the variations in the number of patients and the causes for their admission, we analysed the frequency distribution of patients in the period from 1995 to 2019, as well as the frequency of incidents with them during the months of the year. 

We subjected the survey data to statistical processing (SPSS-Inc 2019). The studied indicators were analysed by means of descriptive statistics (frequency distribution). The significance of the results was represented by the exact P-value (2-tailed), known in SPSS as the Exact Sig (2-tailed). Significance of variables was interpreted at p < 0.05. The obtained results were presented in charts (Excel, Windows 10) and tables.

## Results

According to the analysis of the specialised database of the WRBC for the period between 1995 and 2019, a total of 18,720 patients were registered. Out of all the patients, the majority were birds, accounting for 82.50% (n = 15439). Mammals were the second most common patients, representing 13.00% (n = 2442) and reptiles were the least common with only 4.50% (n = 839, Table 1).

The summarised results showed that 82.22% (n = 15392) of the patients which passed through the Green Balkans Wildlife Rescue Centre fall under at least one of the listed normative acts and/or international instruments that protect their populations.

**Figure 1.**
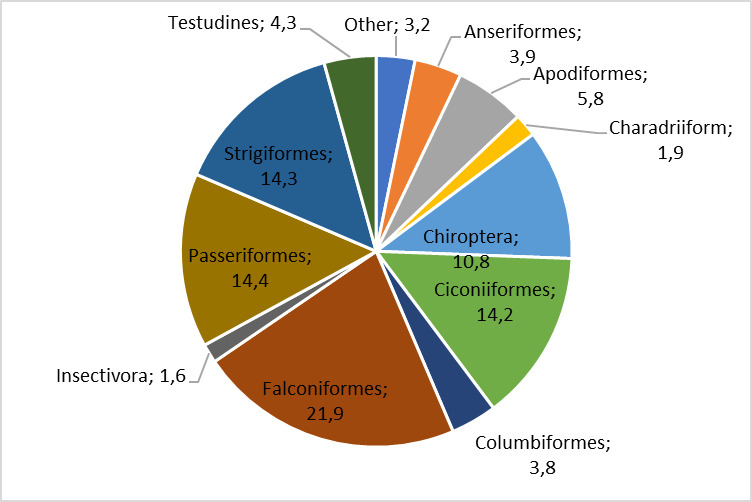
Distribution by order of patients registered at WRBC - Stara Zagora.

Based on our research, we discovered that, between the years 1995 and 2019, over 230 species from 26 different orders were brought to the Green Balkans Wildlife Rescue Centre for treatment. Of those species, 17 were birds, six were mammals and two were reptiles (Fig. 1).

**Figure 2.**
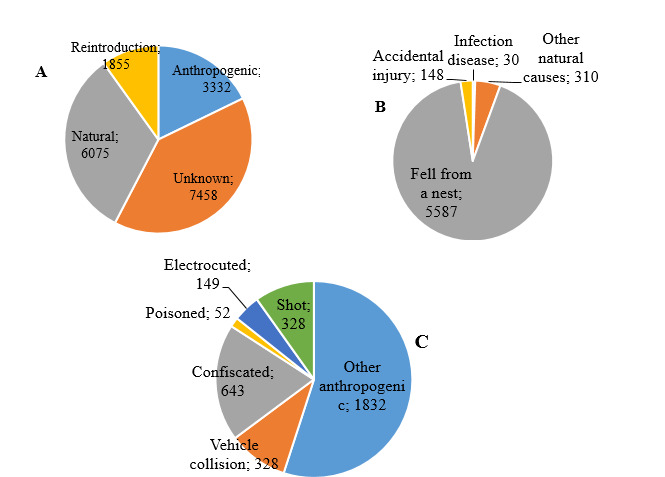
Main causes for admission of wildlife patients to the WRBC in Stara Zagora. **A **Ratio of anthropogenic and natural causes; **B** Etiological factors of a natural origin as a cause for accidents and admission of patients; **C** Etiological factors of anthropogenic nature as a cause for accidents and admission of patients.

The next step in analysing the WRBC-Stara Zagora patient database involved listing the patients according to the cause for their admission. The animals were divided into two main categories: natural causes and incidents involving wild animals resulting from anthropogenic factors (Fig. 2).

The analysis revealed that about 32.5% of all admitted patients had experienced trauma due to natural causes. These natural causes had been further categorised, based on the established etiological factors. Amongst these categories, "Fell from a nest" was found to be the most common reason for admission, accounting for 92.0% of all patients (n = 5587, CI95%: 91.3 ± 92.7). The second most frequent sub-category was "Other natural causes", which included exhaustion, destroyed nest, intraspecific or interspecific conflicts over territory or during the breeding season and attacks by a predator. This category accounted for 5.1% of all patients (n = 310, CI95%: 4.52 ± 5.64).

During the study period, 148 patients (2.4%, CI95%: 2.1 ± 2.8) suffered trauma due to accidental injury or natural disasters, making it the third most common natural cause leading to trauma. Only a small number of patients (n = 30, 0.5%, CI95%: 0.3 ± 0.7) were admitted for viral, bacterial or parasitic infections, which included various diseases such as aspergillosis, trichomoniasis, coccidiosis, endoparasites, E. coli, Salmonella spp. etc. A total of 1,855 patients received treatment and/or prophylaxis during the research period. Amongst them, 10% were wild birds that were part of a re-introduction programme.

In 40% of the cases (n = 7458), the cause of admission for treatment and rehabilitation remained unknown due to a lack of information about the accident.

Anthropogenic causes were a prerequisite for admission to a wildlife rescue centre in 18% (n = 3332) of the recorded cases. In the category "Other anthropogenic factors", there were several factors resulting from human actions that appeared to be a prerequisite for accidents. Such were "killed parents", "entered in a building", "nest destroyed during repairs", "found in a settlement that is atypical for the species", "entangled in rope", "fell in a shaft/pit/oil/", "caught in a trap or snare" etc. Their total number accounted for 1832 specimens [55.0% (CI95%: 53.4 ± 56.7)].

The remaining causes of anthropogenic factors, though less frequent, had a more significant impact on wildlife and biodiversity. These causes included “Confiscated”, which accounted for 643 animals [19.3% (CI95%: 18.0 ± 20.6)]. The “Confiscated”' category involved the confiscation of illegally-bred specimens that were sold in markets, taken away at border inspection posts while being transported for sale abroad and those that had been raised at private properties. When the condition of these animals worsened due to the inability to provide suitable conditions, they were referred to specialists at the Centre for treatment.

During the study period, the third most frequent causes of injury were "Vehicle collision" and "being shot", with 328 patients for each category [9.8% (CI95%: 8.9 ± 10.8)]. The number of patients which were electrocuted throughout the study period was 149 [4.5% (CI95%: 3.8 ± 5.2)]. This included those which were injured in a collision with elements of the power transmission network and those which were directly affected by electric shock. The number of poisoned patients during the study period was 52 [1.6% (CI95%: 1.1 ± 2.0)]. Some of these patients' intoxication was confirmed by laboratory and pathological examinations, while others were counted as such, based on data from anamnesis and clinical signs.

**Figure 3.**
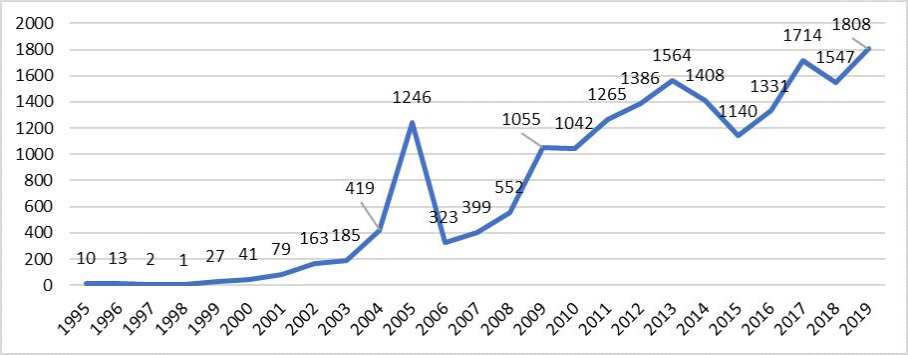
Distribution by year of the patients of WRBC in Stara Zagora for the study period.

According to the frequency distribution by year, the number of patients admitted to the WRBC had gradually increased (Fig. 3). In 2008, there were less than 1,000 injured animals admitted per year, except for 2005 which had 1,246 cases. However, since 2008, more than 1,000 patients have been admitted annually, with the highest number recorded in 2019 at 1,808 (Fig. 3).

**Figure 4.**
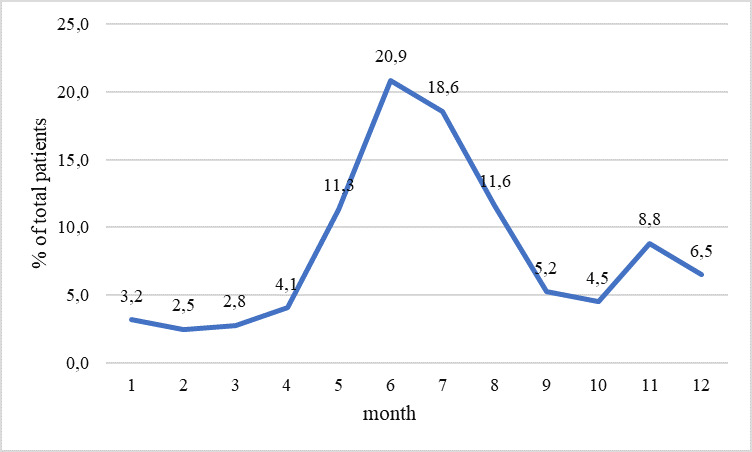
Average distribution of patients admitted per month at the WRBC in Stara Zagora.

Regarding the distribution of cases during the months of the calendar year, a high peak of patient admissions was observed during the summer months. In April, 4.1% (n = 763) of the patients were admitted, while in May, this percentage increased to 11.3% (n = 2123). In June, the highest share of admissions was registered at 20.9% (n = 3474) and this level continued at the same rate until September, with July at 18.6% (n = 474) and August at 11.6% (n = 2176) (Fig. 4).

## Discussion

In many countries, there are established various organisations with similar structures with the aim to protect and rehabilitate wildlife in distress - such as Spain, Italy, Hungary, the USA, Canada, Indonesia, South Africa, New Zealand and Bulgaria. Our data are based on records of a large number of cases treated as patients at the Wildlife Rescue Breeding Centre at Green Balkans-Stara Zagora over a 25-year period. This was the only functioning rescue, rehabilitation and breeding centre in the country during the studied period. In Great Britain, for instance, around 80 wildlife rescue centres have been established (Mullineaux and Kidner 2011), serving approximately 30-40,000 animals per year (Molony et al. 2007). During the research period, the Centre's database recorded 18,720 patients.

For comparison, a retrospective analysis of a rehabilitation centre in Catalonia conducted over a 19-year period included 54,772 cases (Molina-Lopez et al. 2017), while, in northern Portugal, 6,058 patients were examined over a 10-year period. These data placed WRBC at an average position in terms of the number of patients admissions per year compared to other similar centres in Europe. Over 80% of the patients at the Wildlife Rescue Centre were protected under legal national regulations and/or international conventions that aim to safeguard their populations.

This confirms that the ex-situ activity of the WRBC can be used to manage rare species with low population. This is particularly important as each individual's survival is critical for the species' existence. Studies on faunal diversity in central European and Balkan countries showed that there were approximately 30,000 known animal species in Bulgaria, which accounted for 50% of the presumed faunal diversity in the country. So far, the best-studied species were vertebrates, with 800 known species (Golemanski and Popov 2011).

According to literature data (Bunarco 2009), the Class Birds (Aves) represent the highest number of biodiversity amongst vertebrates in our country, consisting of 19 orders out of a total of 52. As a result, it is understandable that 82.50% of all patients admitted for treatment and rehabilitation at WRBC during the study period were birds. Our findings were consistent with those of other rescue centres in Europe where birds constitute the majority of patients (Molina-Lopez et al. 2017, Romero et al. 2019). In one of Italy's largest rehabilitation centres, for example, birds accounted for 80.9% of patients, while mammals made up 18.6% and amphibians were only 0.5% (Dessalvi et al. 2021).

It should be noted that the number of admitted mammals in our study, especially bats, was low due to the operation of a specialised unit for research and treatment of these animals at the Green Balkans Organisation. Hence, only a small proportion of these patients requiring treatment and hospitalisation were referred to the WRBC. Additionally, when considering the percentage of reptiles, it is important to explain that our country has a specialised centre for turtles. These patients were usually transferred to the WRBC after receiving first aid.

The analysis of the reasons for admission of wildlife specimens for therapy in rescue centres generally showed that the factors leading to incidents can be divided into two categories - natural and anthropogenic. Natural factors referred to incidents caused by nature, while anthropogenic factors were due to human activities. Our data on wild birds treated at the WRBC showed that 32.5% of incidents were due to natural causes, while 18% of events were due to anthropogenic factors. Amongst the natural causes, 92% of patients were admitted due to being orphaned or falling from the nest, which is higher than the values reported by Romero et al. (2019) – 4.6%, as well as from Molina-Lopez et al. (Molina-Lopez et al. 2017) – 31.8%. 

Again with higher values compared to the cited authors, we found the patients admitted to the WRBC due to confiscation (illegal possession, including specimens confiscated both at the border and inside the country) accounted for 19.3% of the birds compared to 9.1% in Chile, but were far less than the poached specimens reported for Genoa – 54.3% (Dessalvi et al. 2021), respectively, 39.8% in Catalonia (Molina-Lopez et al. 2017). Ress and Guyer (2004) stated that, of a total of 19 causative agents, the five most common causes of injury to adult birds in the USA were vehicle impact, collision/trauma, shooting, barbed wire entanglement and starvation.

Romero et al. (2019) conducted a study on injured birds in Chile and reported that the leading cause of injuries was illegal possession, which accounted for almost half of all cases and resulted in confiscation in 9.1% of the cases. Orphaning was the cause for 4.6% of the injuries, followed by disease (4.5%), environmental problems (such as falling into oil or being poisoned) for 2.5% and interspecies/intraspecies conflict (such as attacks by dogs, cats or wild animals) for 1.8% of all. Similarly, Rodriguez et al. (2020) analysed the rescue centre's activity in Tenerife and identified vehicle collisions as the primary cause of bird injuries. Other factors included starvation, being caught in sticky traps and being shot.

According to the literature, 14.2% of the birds and animals rehabilitated at the centre in Genoa, Italy, were injured due to human activities, while 54.3% of the hospitalised animals were attacked by predators or poached (Dessalvi et al. 2021). 

According to the analysis of the results of the WRBC's activities, the period from May to August was the busiest time of the year. During this time, about 60% of injured animals were admitted. This peak coincided with the breeding season of birds and the opening of the hunting season for small local and migratory game (Klisurov 2012a). This trend was observed in other centres as well, where the intake of injured wildlife is also associated with the breeding season (Kelly and Bland 2006, Griffiths et al. 2010).

While in the first years of the research period, the inflow of patients was low, with the increase in the popularity and capacity of the WRBC, a natural increase in the number of admitted patients was observed from 1 (1998) to 2 patients per year (1992, 1997), up to over 1000 patients/year (2009 – 2012) (Klisurov 2012b), with the highest number of patients in 2019 – n = 1808. It is hard to explain that, despite several legislative changes in the field of biodiversity, incidents with wild animals have not decreased and their admission to the rescue centre has continued to increase over the years.

 In contrast to other centres in Europe that have maintained a stable number of admitted patients over the years, the WRBC has seen a significant increase in the number of hospitalised animals. For instance, the rescue centre in northern Portugal admitted an average of 700 injured animals yearly between 2009 and 2017 (Garcês et al. 2020). This is believed to be because other countries have adequate rescue centres that can cater for injured animals in their respective areas.

## Conclusions

Wildlife is defined as a natural heritage which we should preserve for the future generations. However, due to many threats arising from natural to anthropological causes, a lot of rare and endangered wild animals are threatened with extinction. In order to preserve biodiversity and protect wildlife, many countries have established wildlife rescue centres, amongst which we have the Wildlife Rehabilitation and Breeding Centre at Green Balkans in Stara Zagora. Being a unique structural and functional unit for protection of wild animals, especially wild birds, the WRBC has treated more than 18000 patients from 1995 to 2019. Amongst all of them, 82.22% (n = 15392) of the registered wildlife fall under at least one of the listed normative acts and/or international instruments that protect their populations.

Our analysis of the WRBC database showed that there is a peak in the number of injured animals during the summer season, mainly due to shooting during the breeding season. At the same time, the most frequent causes amongst the natural factors that led to traumas are classified as “fallen from a nest” for 92.0% of all patients and "other natural causes", including exhaustion, destroyed nest, intraspecific or interspecific conflicts over territory or during the breeding season and attacks by a predator. Meanwhile, the anthropogenic causes that lead to the most traumatic incidents for wildlife were related to “vehicle collision”, “being shot” and “confiscated”. However, in 40% of the cases, the cause of admission to WRBC remained unknown due to a lack of information about the accident.

## Figures and Tables

**Table 1.** Frequency distribution and percentage of patients as per zoological classes and conservation status, which were admitted to the WRBC in Stara Zagora.

**Class**		**Biodiversity act (BG)**	**IUCN**	**BERN**	**CITES**	**BONN**	**Total**
AVES	Count	10923	315	12014	6344	5538	15439
	% of total	58.30%	1.70%	64.20%	33.90%	29.60%	82.50%
MAMMALIA	Count	2239	0	1999	1890	61	2442
	% of total	11.90%	0.00%	10.70%	10.10%	0.30%	13.00%
REPTILIA	Count	798	750	793	750	0	839
	% of total	4.30%	4.00%	4.20%	4.00%	0.00%	4.50%
**TOTAL**	**Count**	**13960**	**1065**	**14806**	**8984**	**5599**	**18720**
	**% of total**	**74.6%**	**5.7%**	**79.1%**	**48.0%**	**29.9%**	**100.0%**
